# Genotype-determined EGFR-RTK heterodimerization and its effects on drug resistance in lung Cancer treatment revealed by molecular dynamics simulations

**DOI:** 10.1186/s12860-021-00358-6

**Published:** 2021-06-10

**Authors:** Mengxu Zhu, Debby D. Wang, Hong Yan

**Affiliations:** Department of Electrical Engineering, City University of Hong Kong, Kowloon, Hong Kong

**Keywords:** Epidermal growth factor receptor (EGFR), Drug resistance, EGFR signaling, Signaling crosstalk, Molecular dynamics (MD) simulations, Geometric properties

## Abstract

**Background:**

Epidermal growth factor receptor (EGFR) and its signaling pathways play a vital role in pathogenesis of lung cancer. By disturbing EGFR signaling, mutations of EGFR may lead to progression of cancer or the emergence of resistance to EGFR-targeted drugs.

**Results:**

We investigated the correlation between EGFR mutations and EGFR-receptor tyrosine kinase (RTK) crosstalk in the signaling network, in order to uncover the drug resistance mechanism induced by EGFR mutations. For several EGFR wild type (WT) or mutated proteins, we measured the EGFR-RTK interactions using several computational methods based on molecular dynamics (MD) simulations, including geometrical characterization of the interfaces and conventional estimation of free energy of binding. Geometrical properties, namely the matching rate of atomic solid angles in the interfaces and center-of-mass distances between interacting atoms, were extracted relying on Alpha Shape modeling. For a couple of RTK partners (c-Met, ErbB2 and IGF-1R), results have shown a looser EGFR-RTK crosstalk for the drug-sensitive EGFR mutant while a tighter crosstalk for the drug-resistant mutant. It guarantees the genotype-determined EGFR-RTK crosstalk, and further proposes a potential drug resistance mechanism by amplified EGFR-RTK crosstalk induced by EGFR mutations.

**Conclusions:**

This study will lead to a deeper understanding of EGFR mutation-induced drug resistance mechanisms and promote the design of innovative drugs.

## Background

Lung cancer is the leading cancer killer globally [1, 2]. Accounting for 85% of all cases, non- small cell lung carcinoma (NSCLC) is a main type of lung cancer and has thus become an active research topic in pathogenesis [3]. Epidermal growth factor receptor (EGFR), belonging to the ErbB family, plays an important role in the molecular pathology of NSCLC and other cancers [4]. Upon activation by a ligand, EGFR will form a homodimer or heterodimer with a partner, which stimulates its tyrosine kinase (TK) activity and switches on the downstream signaling [5, 6]. Over-expression of EGFR appear in nearly 60% of NSCLC patients [7], who frequently harbor EGFR mutations (like L858R) that will lead to aberrant singling, enhanced cell proliferation and progression of lung cancer. Tyrosine kinase inhibitors (TKIs) to EGFR, such as Gefitinib or Erlotinib, are generally effective in treating EGFR-mutated NSCLC patients. However, the treatment will encounter failures later due to the emergence of drug resistance [8, 9]. Well-explained reasons for such resistance lie in the decreased EGFR-TKI binding affinity induced by a second EGFR mutation (like T790M, accounting for 50% of resistant cases) [10–13] and the associated alterations in cell signaling pathways caused by crosstalk between EGFR and other receptor tyrosine kinases (RTKs) [14, 15].

Crosstalk between EGFR and other RTKs, such as ErbB-family members [16], c-Met [15] and Insulin-like growth factor 1 receptor (IGF-1R) [17, 18], are vital in ascertaining the mechanisms of drug resistance in cancer treatment. Previous research showed that both direct and indirect connections exist, including direct heterodimerization with EGFR to participate in the cell signaling [19–22]. For c-Met specifically, Tanizaki et.al have revealed that Met can form heterodimers with ErbB family members with immunoprecipitation [22]. Wheeler et.al also showed that EGFR heterodimerized with cMet [23]. Harwardt et al. and Knowles et al. have found the presence of heteromeric complex of EGFR and cMet by microscopy [24] or antibody array method [25]. Besides, Ortiz-Zapater et al. studied EGFR-Met dimerization on cell lines [21], and Lee et al. applied co-immunoprecipitation and Forster resonance energy transfer (FRET) FLIM to study the EGFR-Met dimers [26]. For IGF-1R, Nahta et al. have studied the influence of IGF-1R/HER-2(ErbB-2) dimers to drug resistance by experiments on cells [27]. Iyer et al. studied the heterodimerization of EGFR with IGF-1R on head and neck cancer cells [28]. Additionally, Oliveira et al. proposed that both direct and indirect crosstalk exists between EGFR and IGF-1R, from association through signal network to direct dimerization [29]. Morgillo et.al studied the drug resistance related to IGFR/EGFR heterodimer on lung cancer cell lines and mice [30]. Becker et.al also investigated EGFR/IGFR dimers in non-small-cell lung cancer (NSCLC) cell lines [31]. EGFR-targeted TKIs cannot block the interactions (heterodimerization) between EGFR and other RTKs, and such unblocked interactions can even be amplified due to EGFR mutations. The mutation-induced amplified crosstalk or signaling will potentially lead to drug resistance. Assisted by a c-Met TKI (SGX523), Ortiz-Zapater et al. have assessed cell proliferation in vitro, tumor growth and EGFR-c-Met dimerization for lung cancer cell lines with different EGFR mutations. They showed significantly reduced cell proliferation, tumor growth and EGFR-c-Met dimerization caused by SGX523 in the resistant mutation case (L858R-T790M) than the other cases [21]. This demonstrated an amplified heterodimerization between c-Met and the resistant EGFR mutants than non-resistant ones, which further implies that EGFR-c-Met heterodimerization is determined by EGFR genotype [21]. Dual inhibitors to both EGFR and c-Met can mitigate the drug-resistant cases [32, 33], which also demonstrates the enhanced EGFR-c-Met heterodimerization as a potential resistant mechanism. Such mechanism also applies to the crosstalk between EGFR and IGF-1R [29, 30, 34]. Clinical studies have shown that IGF-1R-targeted TKIs can inhibit NSCLC cells that are resist to EGFR-targeted drugs [30], implying the significant role of IGF-1R in drug resistance mechanisms. In this study, we aim to explore the EGFR-mutation induced drug resistance mechanism through the perspective of EGFR-RTK crosstalk (heterodimerization), and we considered ErbB2 (amplification frequently occurs in NSCLC), c-Met and IGF1-R as partners for mutated EGFRs.

Molecular structural analysis, dynamics simulations and computational characterization have facilitated a great number of studies of EGFR-mutated lung cancer [16, 35–41]. Specifically, we studied the interactions between mutated EGFRs and an RTK partner (ErbB2, c-Met or IGF1-R) in heterodimerization using such computational techniques. Wild type (WT) EGFR, EGFR with the TKI-sensitive mutation L858R (point mutation at exon 21) [42] and that with co-expressed L858R and T790M mutations (TKI-resistant) [43] were comparably investigated in this study. Molecular dynamics (MD) simulations, as a widely used method in researching about lung cancer [40, 41, 44], were implemented on EGFR-RTK heterodimer structures, and three- dimensional Alpha Shape modeling was subsequently applied to each MD trajectory frame for extraction of geometrical properties [45, 46]. Based on the simulation and modeling results, we characterized the EGFR-RTK interactions in the heterodimers by geometrical properties of the interaction sites, including matching rate of atomic solid angles in these sites and center-of- mass distances between interacting atoms. Computationally estimating the free energy of binding has been broadly used in revealing molecular binding affinities in previous studies [40, 41, 47]. As a benchmark method, it was also applied in this study to validate the geometrical characterization. These characterization methods will shed light on the correlations between the genotypes and the EGFR-RTK heterodimerization, further providing a deeper understanding of the mutation-induced drug resistance mechanisms.

## Results

### Modeling of EGFR mutants, formation of EGFR-RTK heterodimers and MD simulations of heterodimers

**Fig. 1:**
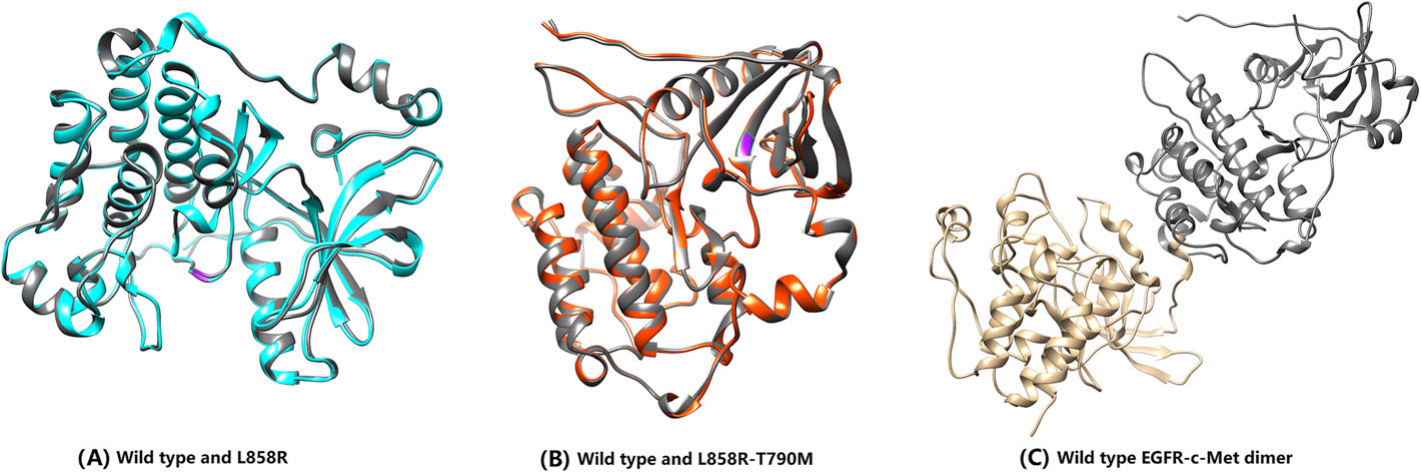
(**a**) Modeled L858R (blue) mutant and wild type (grey) EGFR structures, with the mutation site colored purple. (**b**) Modeled L858R-T790M (red) mutant and wild type (grey) EGFR structures, with the mutation sites colored purple. (**c**) WT EGFR-c-Met heterodimer structure (WT EGFR: grey, c- Met: gold)

To derive the structures of EGFR mutants (L858R and L858R-T790M), we adopted *Rosetta* to model such structures according to homology modeling techniques. WT EGFR was used in this modeling as the structural template, whose unresolved residues were generated using *Rosetta* prior to the mutant modeling. The modeling results are shown in Fig. 1a and b, where the WT protein is superimposed on the mutant structures to shown the mutation sites. To form the EGFR-RTK heterodimer structures, we used the WT EGFR-EGFR homodimer structure as a template and aligned an EGFR WT/mutant (WT, L858R or L858R-T790M) and an RTK (c-Met, ErbB2 or IGF-1R) to the two positions in the dimer. An example of such heterodimers, namely the WT EGFR-c-Met dimer, is presented in Fig. 1c.

For each heterodimer, we simulated its dynamics in explicit-solvent environment using *Amber* software suite. A series of procedures were sequentially imposed on the solvated system, including a short energy minimization, a heating process, and a number of equilibration steps. An equilibrated system can guarantee a reliable production MD simulation for our analysis.

**Fig. 2:**
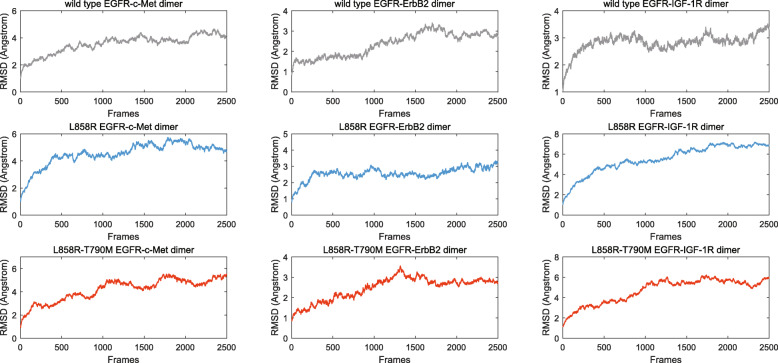
RMSD curves of the EGFR-RTK heterodimers, referring to the first frame, in the equilibration phase of the MD simulations

Therefore, the equilibration of each system was verified prior to the production MD step, using the root-mean-square-deviation (RMSD) curve of the EGFR-RTK dimer in the equilibration phase. Such curves for the heterodimers (250 frames at time interval of 2 ps) are shown in Fig. 2, where each equilibration can be verified by a stable curve. Our production MD simulations for each heterodimer lasted for 3 ns, resulting in a trajectory of 5000 frames (interval of 10 ps).

### Extraction of geometrical properties of EGFR-RTK interfaces in heterodimers and investigation of drug resistance mechanisms

**Fig. 3:**
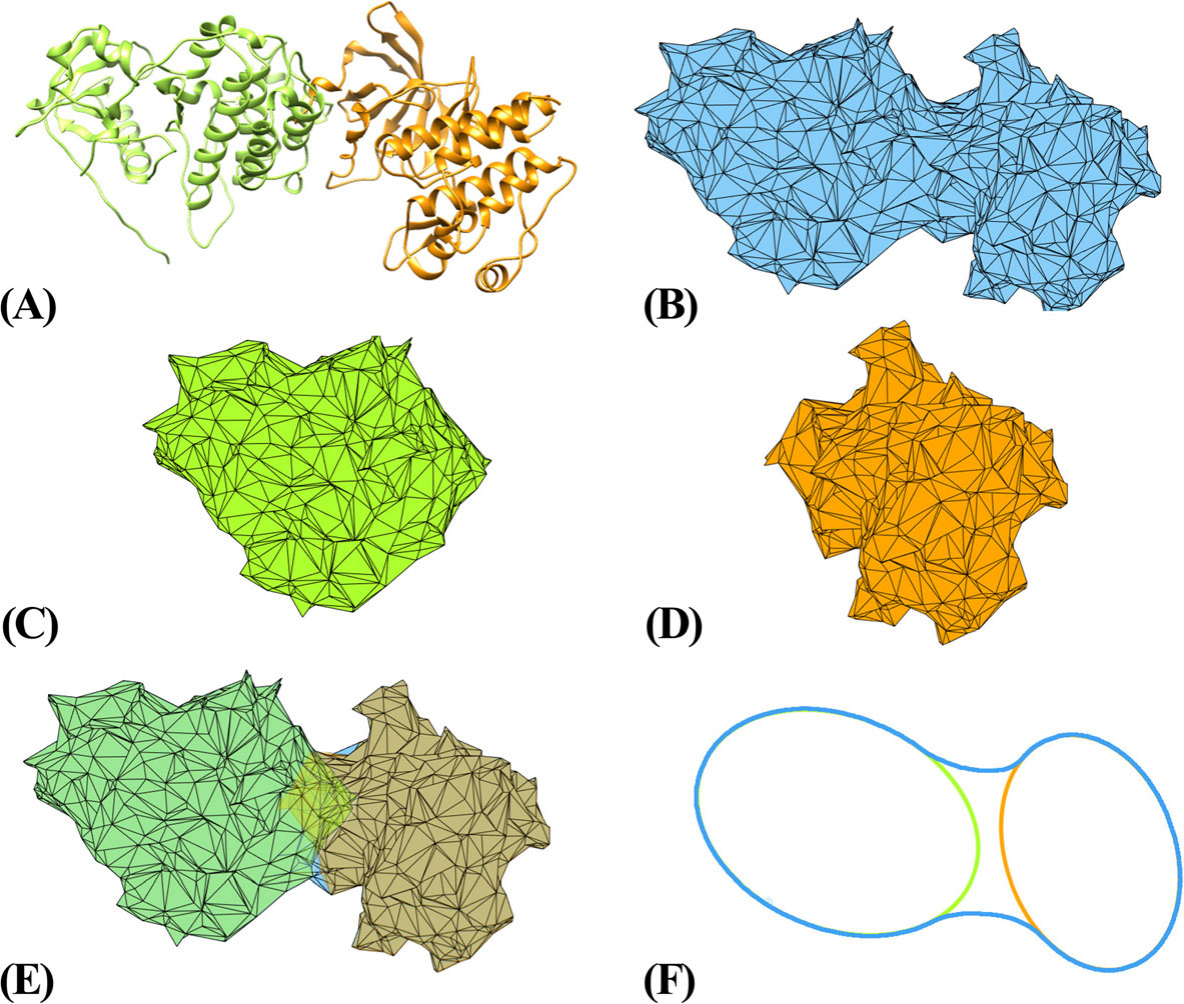
Procedures to capture the interfacial atoms

As we are more interested in the EGFR-RTK interactions in each heterodimer in the dynamics, we first extracted their interfaces using weighted Alpha Shape modeling for all the MD trajectory frames based on Eq. (5). As an example, procedures to generate interfacial atoms of the WT EGFR-c-Met dimer in one trajectory frame (randomly selected) are showed in Fig. 3.

Sub-Fig. A is the original wild type EGFR-cMet dimer. We applied Alpha Shape Modeling to reconstruct the surface of the dimer (subfigure B). Similarly, we reconstructed the surfaces of the two proteins in the dimer (subfigure C: EGFR, subfigure D: cMet). The surface atoms of EGFR that are not on the surface of the dimer (comparing subfigures B and C) are regarded as the interfacial atoms on EGFR, and similarly we can capture the interfacial atoms on cMet (comparing subfigures B and D). Subfigures E and F show the main idea of this process.

**Fig. 4:**
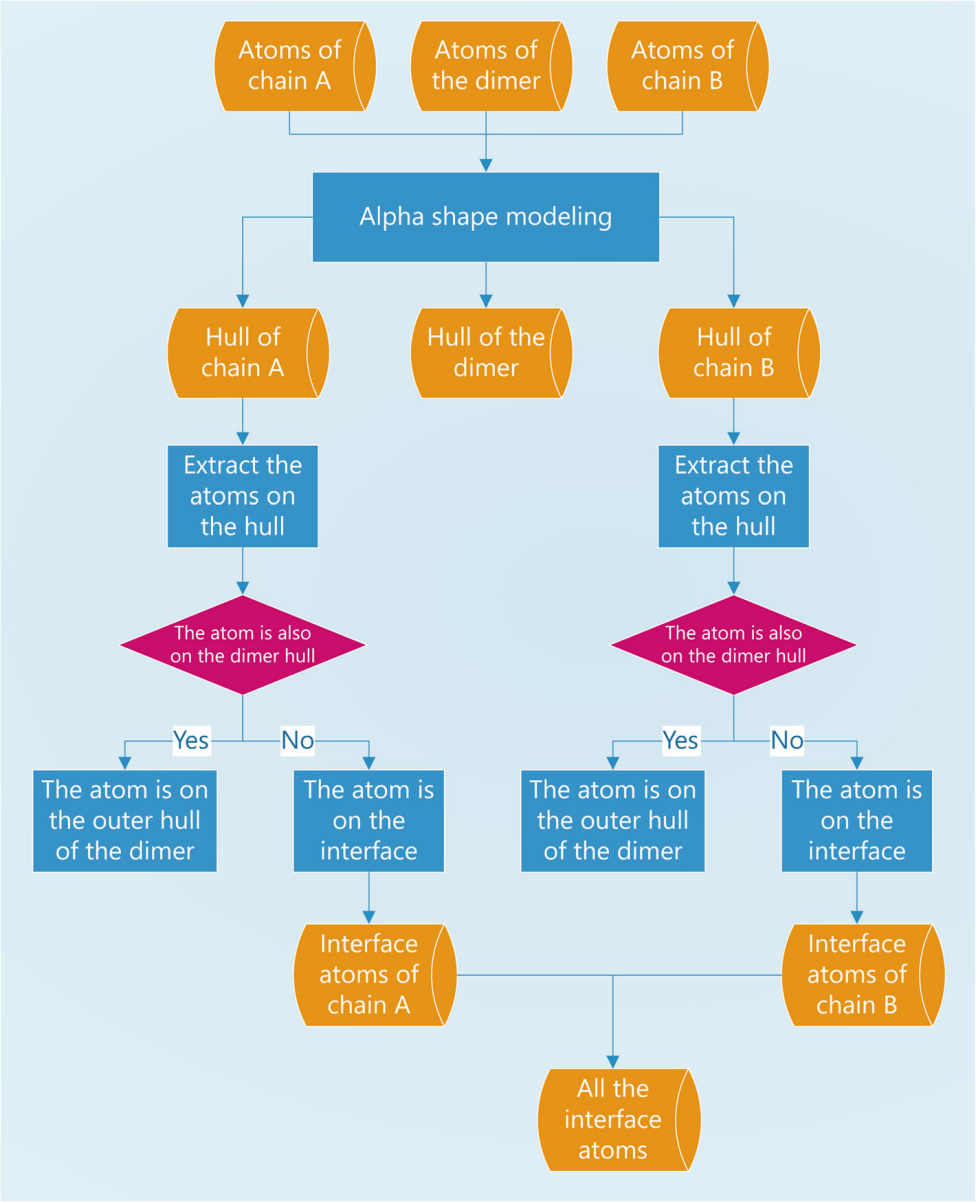
Flow chart of the algorithm to obtain the interface atoms

The procedures are shown in the following diagram, as in Fig. 4. Information about the resulting interfacial atoms in this example is listed in Table 1.

**Table 1:** Information of the interfacial atoms. Indices of the atoms and residues here are different form the numbers in the original pdb file, as they were renumbered sequentially from 1.

Chain label	Residue number	Residue name	Atom number	atom type	interacting partners
A	1	GLN	3	C	3862,4094,4098
A	1	GLN	8	C	4098,4229
A	2	ALA	11	C	4097,4098
A	2	ALA	13	C	4200
A	5	HIE	34	C	3838,3860,3861
A	7	VAL	50	C	3838,4222,4224
A	56	GLN	427	C	4221
A	56	GLN	428	O	4221
A	56	GLN	431	C	
A	59	THR	456	C	4199
A	60	GLU	458	N	4199
A	60	GLU	459	C	4199,4176
A	60	GLU	461	C	4199
A	62	ILE	474	C	4176,4199,4200
A	62	ILE	475	C	4194
A	62	ILE	477	C	
A	62	ILE	478	O	4194
A	80	LEU	610	C	4073
B	481	VAL	3838	C	34,50
B	484	TRP	3860	C	34
B	484	TRP	3861	C	34
B	484	TRP	3862	C	3
B	511	ARG	4073	C	610
B	512	LEU	4080	C	
B	514	GLN	4094	C	3
B	514	GLN	4097	C	11
B	514	GLN	4098	O	3,8,11
B	523	TYR	4171	O	
B	524	MET	4176	S	459,474
B	526	MET	4194	C	475,478
B	527	VAL	4196	N	
B	527	VAL	4199	C	456,458,459,461,474
B	527	VAL	4200	C	13,474
B	530	TRP	4221	C	427,428
B	530	TRP	4222	C	50
B	530	TRP	4224	C	50
B	530	TRP	4229	C	8

For each heterodimer, we kept the pairs of interfacial atoms sharing an edge in any tetrahedron that connects the interaction sites of the two chains, and calculated the average solid angles of these interfacial atoms of each trajectory frame (Eq. (6)). Subsequently, the matching rate, namely the ratio of matched solid angle pairs over all pairs, was derived according to Eq. (7). Comparing the WT EGFR and mutant L858R, the matching rate along the MD trajectory for each of the EGFR-RTK heterodimers is displayed in Figs. 4a (EGFR-c-Met), 5B (EGFR- ErbB2) and 5C (EGFR-IGF-1R) respectively.

**Fig. 5:**
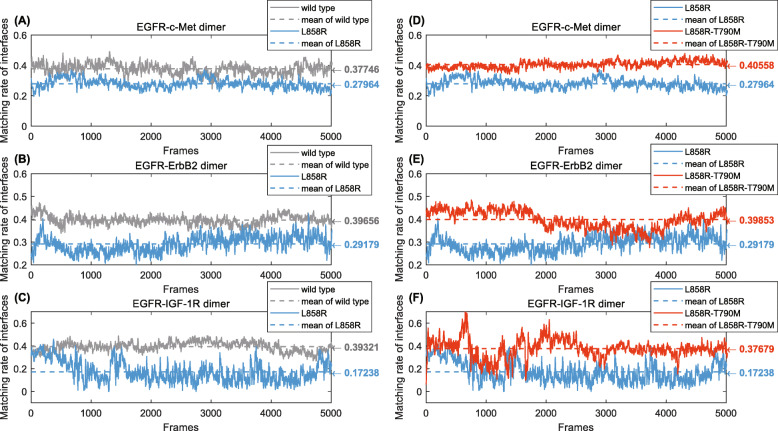
Matching rate of interfacial atoms along the MD trajectory for EGFR-RTK heterodimers (smoothed by moving-averaging), with the average for each curve shown as a dashed line. (**a**) Comparison between WT EGFR-c-Met and L858R-c-Met dimers. (**b**) Comparison between WT EGFR-ErbB2 and L858R- ErbB2 dimers. (**c**) Comparison between WT EGFR-IGF-1R and L858R- IGF-1R dimers. (**d**) Comparison between L858R- c-Met and L858R-T790M-c-Met dimers. (**e**) Comparison between L858R-ErbB2 and L858R-T790M- ErbB2 dimers. (**f**) Comparison between L858R-IGF-1R and L858R-T790M- IGF-1R dimers

Furthermore, we calculated the average COM distance of interfacial atom pairs, each of which share an edge in any boundary tetrahedron, for each EGFR-RTK heterodimer along the MD trajectory. Comparisons of the COM distance trajectories are displayed in Fig. 5.

**Fig. 6:**
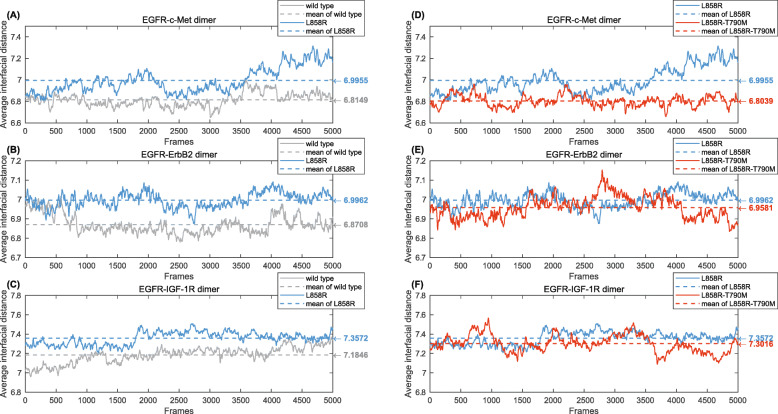
Average center-of-mass distance of interfacial atom pairs along the MD trajectory for EGFR- RTK heterodimers (smoothed by moving-averaging), with the mean for each curve shown as a dashed line. (**a**) Comparison between WT EGFR-c-Met and L858R-c-Met dimers. (**b**) Comparison between WT EGFR-ErbB2 and L858R- ErbB2 dimers. (**c**) Comparison between WT EGFR-IGF-1R and L858R- IGF-1R dimers. (**d**) Comparison between L858R-c-Met and L858R-T790M-c-Met dimers. (**e**) Comparison between L858R -ErbB2 and L858R-T790M-ErbB2 dimers. (**f**) Comparison between L858R-IGF-1R and L858R-T790M- IGF-1R dimers

Finally, we estimated the binding free energy within each EGFR-RTK heterodimer using MM/GBSA in *Amber*. For each RTK partner (c-Met, ErbB2 or IGF-1R), WT EGFR, L858R and L858R-T790M were compared as a group in Fig. 6.

## Discussions

The methods revealed properties of the dimers. As shown in Fig. 5, L858R-RTK dimers generally have a lower matching rate of interfacial atoms than WT EGFR-RTK dimers. Such differences indicate a looser crosstalk between L858R and downstream RTKs, than that between WT EGFR and RTKs. It demonstrates that for TKI blocked EGFR with L858R mutation there is a looser crosstalk between EGFR and RTKs, guaranteeing the effectiveness of EGFR-TKIs in treating such mutated cases in the perspective of signaling crosstalk. The comparison results of matching degree for L858R-RTK and L858R-T790M- RTK heterodimers are presented in Figs. 5d-f. Compared to L858R, mutation L858R- T790M corresponds to a higher matching rate for all the EGFR-RTK dimers, implying a tighter crosstalk between L858R-T790M and these RTKs than that between L858R and RTKs. This amplified crosstalk for mutant L858R-T790M probably induces the drug resistance, compared to L858R.

The COM distance method shows similar results. For WT EGFR and mutant L858R, distance trajectory for each of the EGFR-RTK heterodimers is presented in Figs. 6a-c respectively. L858R-RTK dimers generally have a longer interfacial distance than the WT EGFR-RTK dimers, implying a higher instability and a looser crosstalk. Similarly, comparisons between L858R-RTK dimers and L858R-T790M- RTK dimers in Figs. 6d-f show a shorter interfacial distance and a tighter crosstalk between L858R-T790M and RTKs. This proposes the effectiveness of TKIs to mutation L858R and the resistance mechanism of TKIs to a second mutation T790M, from the perspective of heterodimer stability and EGFR-RTK crosstalk.

**Fig. 7:**
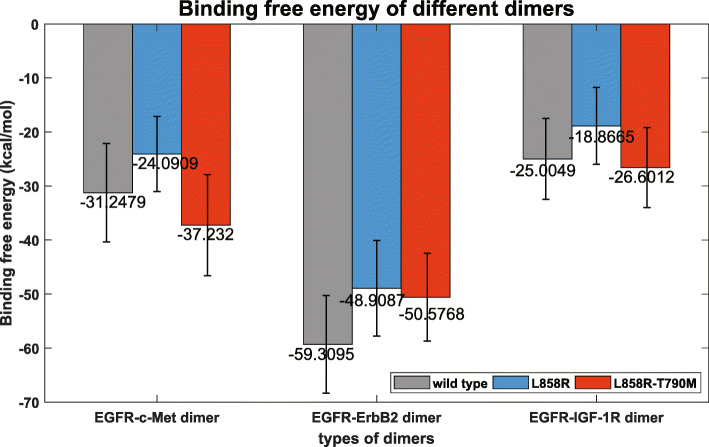
Computational binding free energies of EGFR-RTK heterodimers. EGFR variants include wild type EGFR, mutant L858R and mutant L858R-T790M. RTK partners include c-Met, ErbB2 and IGF- 1R. Bar heights indicate the average binding free energies of these heterodimers, and the errors for each bar are also presented

Results of binding free energy method provides another evidence. As shown in Fig. 7, for all RTK partners, L858R corresponds to the lowest binding affinity with them (highest binding free energy), showing the effectiveness of TKIs to this mutation. Comparing to L858R, a second mutation T790M (co-expressed L858R and T790M mutations) results in a higher binding affinity (lower binding free energy) with the RTKs, promisingly indicating the drug resistance mechanism of a tighter L858R-T790M-RTK crosstalk. These results are consistent to those revealed by the interfacial geometrical properties that are extracted in the preceding sections.

## Conclusion

Protein mutations will result in deformation of protein surfaces and changes in geometrical property, which will alter their interactions with other partners and thus modify their functions. In this study, we examined how mutation influences the crosstalk between EGFR, who plays a significant role in lung cancer progression, and its RTK partners in downstream signaling pathways. WT EGFR, a TKI-effective mutation L858R and a TKI-resistant mutation L858R-T790M were considered and paired up with RTK partners c-Met, ErbB2 and IGF-1R to form EGFR-RKT heterodimers. MD simulations were implemented for each dimer to uncover its dynamics, after which Alpha Shape modeling was applied to reveal the geometrical properties of dimer interfaces. Specifically, matching rate of atomic solid angles in the interaction sites and center-of-mass distances between interacting atoms of each dimer were calculated based on the extracted Alpha Shapes. Results show that L858R is the most alienated from the RTK partners while a second mutation (T790M) will make it closer to the RTKs. This demonstrates a potential drug resistance mechanism which is resulted from the amplified EGFR-RTK crosstalk induced by EGFR mutations. It also shows that such EGFR-RTK heterodimerization is determined by EGFR genotypes. Conventional estimation of binding affinity for a molecular binding system based on MD simulations, namely calculating the free energy of binding, was also implemented to support the study. Consistent results were derived according to such conventional methods, which guaranteed the finding revealed by the geometrical characterization.

This study will lead to a deeper understanding of EGFR mutation-induced drug resistance in lung cancer treatments and will promote the design of genotype-determined therapies or drugs. Our work also has limitations. Although our results are self-consistent and agree with earlier experiments by other researchers, this work is based on computer simulations only. In further research, experimental methods are necessary to further validate the results and reveal the mechanism of drug resistance. We hope this work can provide theoretical guidance for future experiments and clinical applications. Moreover, the geometrical characterization of molecular interfaces proposed in this study will facilitate other studies for feature extraction or interface modeling.

## Methods

### Modeling EGFR mutants and constructing EGFR-RTK heterodimers

The crystal structure of a homodimer of WT EGFR tyrosine kinase (TK) domains was collected from the Protein Data Bank (PDB) [48, 49]. PDB:2GS2, an X-ray structure with resolution of 2.80 Å was selected as template. In what follows, we abbreviated the TK domain to simplify the presentation. Residues at positions 672 to 994 on chain A of 2GS2 in original PDB file were selected and represented as indices 1–323 for following procedures. *Rosetta* was used to model the L858R and L858R-T790M mutant structures using the monomer WT EGFR as a template [50]. Some regions on the structure are not resolved in the X-ray diffraction, and residues in these regions do not have detailed coordinates in the original PDB file. For PDB:2GS2, residues at positions 723–725 and 967–981 in the original PDB file are unresolved in the crystal structure. Thus, prior to the modeling, we generated the coordinates of the unresolved residues in the crystal structure according to *Rosetta comparative modeling* (CM) protocol. To achieve this, we prepared the fragment files based on the fragment library in *Rosetta*, [51] where proteins can decompose into 9mer and 3mer fragment files. *Rosetta* will rank the generated structures according to the energy, a lower energy corresponds to a more stable state. Our refined template structure (with coordinates of unresolved residues generated) was derived based on this principle (*extract_pdb* protocol in *Rosetta*). Then we use the high- resolution *ddg_monomer* (HRDM) protocol to generate the structures of mutants L858R and L858R-T790M, where L858R means a point mutation at the 858th position from Leucine (L) to Arginine (R) and T790M represents a replacement from Threonine (T) to Methionine (M) at the 790th position. In such modeling, the Harmonic distance constraints were applied for pre- minimization, and all the C-alpha atoms in both backbone and side chains were restricted within 9 Angstroms. Default parameters in *score12* of *Rosetta* were used. Weights of repulsive term were increased to 100% with three rounds of minimization executed.

Early research by Zhang et al. [49] showed that a damaged/blocked EGFR can be re-activated in the signaling pathway if there are an intact C-lobe in EGFR and an RTK-partner with an intact N-lobe/ATP site. Accordingly, we hypothesize that one possible drug-resistance mechanism for a drug-blocked EGFR is providing its C-lobe face to interact with the N-lobe faces of other RTK partners (re-activation of EGFR signaling). Driven by this idea, we attempted to form the EGFR-partner dimers first in our simulation studies. As crystal structures of these dimers are not available, we modeled them using a template of similar structures and the homology modeling techniques. The computational methods applied here could bring more uncertainty. Thus, we used mature techniques that have played an important role in virtual screening and drug design. The structures produced using proper templates and these techniques have been broadly applied in molecular and structural biology research, and proved to be reliable.

Referring to the asymmetric EGFR-EGFR dimer structure, the “Align” function of Chimera [52] was employed. Based on sequence-alignments and coordinate-superposing methods, we superpose the structure on the template, and generate the new coordinates to achieve spatial proximities. In this study, we used the ErbB2 homodimer PDB ID: 3PP0 [53], which provide a crystal structure with X-ray diffraction at the resolution of 2.25 Å as the template. In PDB:3PP0, N-lobe of chain A faces the C-lobe of chain B. Thus, the RTK structures were superposed to chain A of the 3PP0 template using the “align” function to provide the N-lobe for interactions, and the EGFR variants (wild-type and mutants) structures were superposed to chain B of 3PP0 to provide the C-lobe. The C-Met structure applied here is from PDB ID:4GG5(2.42 Å resolution) [54], and the IGF-1R structure is from PDB ID:5FXQ (2.30 Å resolution) [55]. Both crystal structures were obtained from X-ray diffraction. Each modeled EGFR-RTK dimer structure was then retained for subsequent energy-minimizations and MD simulations.

### Molecular dynamics (MD) simulations

MD simulations are a popular method for studying the physical movements of molecules at the atomic level. The MD simulator considers the initial states, force fields and potential energies in the system, and solves the Newton’s equations of motions for every atom based on the known forces and energy [56]. Positions of atoms are updated and recorded at every time step. The position trajectories can provide valuable information about the behavior and functions of molecules. In this study, we performed MD simulations on each EGFR-RTK heterodimer structure using Amber software suite [57], based on the explicit-solvent model. *Ff99SB* and *gaff* force fields were adopted in the simulations. The dimers were computationally solvated within periodic water boxes of TIP3P water model. The minimal distance between each dimer and the walls of its belonging water box was set to 10 Å to achieve efficient simulations. The solvated system was neutralized using Na + and Cl- ions. Such settings were adopted by the tleap module of Amber. After setting up the environment, each system was first energy-minimized by 20,000 cycles prior to the simulations. Then each system underwent the heating (100 ps), density equilibration (100 ps) and constant-pressure equilibration (5 ns) processes before the production simulation. The ultimate production simulations on the equilibrated structures lasted for 50 ns (ns), each resulting a trajectory of 5000 frames for subsequent analysis.

### Characterization of EGFR-RTK interaction in a dimer

#### Alpha shape modeling and matching rate of interfacial atoms

**Fig. 8:**
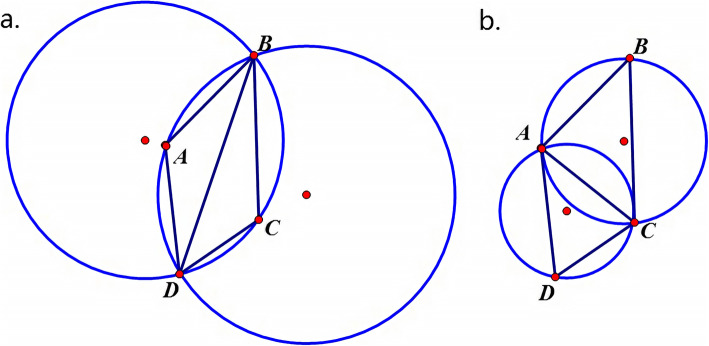
Two examples of triangulation of a point set {**a**, **b**, **c**, **d**}. (**a**) Triangulation that does not meet the requirements of Alpha Shape modeling. (**b**) Triangulation that fulfils the requirements of Alpha Shape modeling

Alpha Shape modeling is actually a linear approximation method, which reconstructs the surface of an object and reveals its geometrical properties effectively [58]. The theory of Alpha Shape modeling originates from triangulation algorithms. The basic criterion of a triangulation for a set of point is that no point in the set will be in the circumcircle of any triangles from the triangulation. Figure 8 shows two simple examples of triangulation for a point set {**A**, **B**, **C**, **D**}, where one of them fulfils the criterion and the other does not.

Algorithms are designed to accomplish the idea. One of the most popular methods is the Delaunay triangulation, whose core idea is to maximize the minimum of all angles in the simplexes based on following determinant [59]:

1 $$ \left|\begin{array}{cc}\begin{array}{cc}{x}_A& {y}_A\\ {}{x}_B& {y}_B\end{array}& \begin{array}{cc}{x}_A^2+{y}_A^2& 1\\ {}{x}_B^2+{y}_B^2& 1\end{array}\\ {}\begin{array}{cc}{x}_C& {y}_C\\ {}{x}_D& {y}_D\end{array}& \begin{array}{cc}{x}_C^2+{y}_C^2& 1\\ {}{x}_D^2+{y}_D^2& 1\end{array}\end{array}\right|>0 $$

where (*xi*, *yi*) represent the coordinates of a point, **A**, **B**, **C** or **D**. A positive value of this determinant indicates that point **D** is in the circumcircle of triangle **ABC**. After implementing 3D Delaunay triangulation, the circumscribed sphere of every tetrahedron can be calculated by Alpha Shape algorithm. The algorithm checks the circumscribed spheres of the tetrahedrons so that squared radii of the spheres are smaller than or equal to a predefined value *α* and the spheres contain no points in the target set. Ultimately, the corresponding tetrahedrons of qualified spheres form an alpha shape.

In this study, we consider all the heavy atoms in a dimer as the point set to be modeled. To simulate the differences of mass between various kinds of atoms, we adopted the weighted Alpha Shape method that takes both location and weight into consideration [45]. Here we define two points, ${p}_1=\left({p}_1^{\prime },{p}_1^{\prime \prime}\right)$ and ${p}_2=\left({p}_2^{\prime },{p}_2^{\prime \prime}\right)$, for instance, where ${p}_1^{\prime }$ and ${p}_2^{\prime }$ represent the locations, and and stand for weights of the points. We define $dist\left({p}_1^{\prime },{p}_2^{\prime}\right)$ as the Euclidean distance between the two points, then the algorithm compares this distance and the sum of weights as follows:

2 $$ dist\left({p}_1^{\prime },{p}_2^{\prime}\right)={p}_1^{\prime \prime }+{p}_2^{\prime \prime } $$

3 $$ dist\left({p}_1^{\prime },{p}_2^{\prime}\right)>{p}_1^{\prime \prime }+{p}_2^{\prime \prime } $$

Equation 2 guarantees two orthogonal points and eq. 3 two sub-orthogonal points. For a predefined real value *α*, a point can be defined as:

4 $$ {P}_{\alpha }=\left({p}^{\prime },{p}^{\prime \prime }+\alpha \right) $$

For a satisfied weighted Alpha Shape, each tetrahedrons has a point that is orthogonal to the points that are relevant to the vertices of the tetrahedron, and sub-orthogonal to all the other points. In this study, we employed the Computational Geometry Algorithm Library (CGAL) to compute weighted Alpha Shape models of each MD frame [60]. Value of α was set as 0, and weights of atoms were defined as the square values of their Van der Waals radii.

We are more interested in the atoms at the interaction sites in a dimer. Alpha Shape modeling reveals the atoms on the geometrical surface of a protein or complex, and we derived the interfacial atoms as follows. First, we extracted the surface atoms of a dimer, defined as point set M, using the method. Then we calculated the Alpha Shapes of each monomer EGFR or RTK individually, which resulted surface point sets A and B. The interfacial atom set X, coupled with those for the individual chains (XA and XB), can be derived according to the following equation:

5 $$ \left\{\begin{array}{c}X=\left(A\cup B\right)-M\\ {}{X}_A=X\cap A\\ {}{X}_B=X\cap B\end{array}\right. $$

To characterize the interactions in a dimer based on the extracted Alpha Shapes, the convex/concave degree of interfacial atoms, which can simply be indicated by solid angles, were used [61]. For a tetrahedron with vertices **A**, **B**, **C** and **D**, the solid angle of **A** can be expressed by the dihedral angle between triangle ABC and ABD (φ_퐴퐵_), that between triangle ABC and ACD (φ_AB_), and that between triangle ABD and ACD (φ_AB_). The overall solid angle is then expressed in Eq. (8), where index *i* represents any tetrahedron that is topped with vertex **A** and the sum of values for all tetrahedrons is finally rescaled into the rage of [− 1, 1]. Ω indicates the average curvature at vertex **A**, with a positive value showing a convex shape and a negative value a concave shape, defined as

6 $$ {\Omega}_A=\cos \left(\frac{\sum_i\left({\varphi}_i^{AC}+{\varphi}_i^{AB}+{\varphi}_i^{AD}-\pi \right)}{4}\right) $$

Subsequently, the interactions in dimers can be estimated by the matching rate of such atomic solid angles at the interaction sites of the two chains. For a pair of atoms (**A** and **B**) sharing an edge in any tetrahedron that connects the two interaction sites, we calculated their solid angles (Ω_A_ and Ω_B_). Then whether the pair of atoms are matched or not, (*A*, *B*), can be inferred according to the following equation:

7 $$ f\left(A,B\right)=\left\{\begin{array}{c}1,{\Omega}_A\times {\Omega}_{\mathrm{B}}<0\\ {}\ 0, otherwise\ \end{array}\right. $$

A matched pair means a pair of convex-concave complementary atoms, while an unmatched pair means the two atoms both have either convex solids or concave ones. For each EGFR- RTK dimer, we calculated the matching rate of the two chains, namely the ratio of matched pairs over all pairs ∑_(*i*, *j*)_*f*(*A*_*i*_, *B*_*j*_)/*N*, along the MD trajectory to show the interactions.

#### Center-of-mass distances between interfacial atoms

As stated above, each pair of atoms (**A** and **B**) sharing an edge in any tetrahedron that connects the two interaction sites were considered in this study. Specifically, we calculated the center- of-mass (COM) distance of each pair (*d*_*COM*_(*A*, *B*)) . Locations of mass centers of the atoms are provided by *cpptraj* tool in *Amber* [62]*.* The center of mass for a series of particles (masses of *x*_1_, …, *x*_*N*_) along a specific coordinate axis (푥) can be simply expressed as follows:

8 $$ {X}_{COM}=\frac{\sum_{i=1}^N{m}_i{x}_i}{\sum_{i=1}^N{m}_i} $$

For all pairs of atoms connecting the two chains in a dimer, we averaged their COM distances $\overline{\left({d}_{COM}\left(A,B\right)\right)}$ and monitored such averaged distance among the MD trajectories. Such simple metric can measure the interaction between the two chains in the dimer and thus reveal the stability of the whole binding system.

#### Free energy of binding for EGFR-RTK dimers

Conventional computational methods to measure the binding strength of a system include the calculation of free energy of binding, and here we also applied such methods in our study. Free energy of binding is normally calculated based on the thermodynamic cycle, where the energy itself in a solvent environment is obtained indirectly as follows [63]:

9 $$ \Delta  {G}_{bind}=\Delta  {G}_{sol, bind}-\Delta  {G}_{vac, bind}=\Delta  {G}_{sol, dimer}-\Delta  {G}_{sol, chainA}-\Delta  {G}_{sol, chainB} $$

Here, ∆*G*_*sol*, *bind*_ is the energy difference between bounded and unbounded states in a solvent environment, while ∆*G*_*vac*, *bind*_ is the difference in the vacuum environment. ∆*G*_*sol*, *dimer*_, ∆*G*_*sol*, *chainA*_ and ∆*G*_*sol*, *chainB*_ are solvation free energies of the dimer, chain A and chain B respectively. A lower value (negative) of binding free energy represents a tighter binding affinity within the binding system. Molecular mechanics generalized Born and surface area (MM/GBSA) continuum solvation is a typical approach for estimating the binding free energy, and we applied such protocol in *Amber* to estimate the interaction strength in different EGFR- RTK heterodimers and to fertilize the geometrical characterization in preceding sections.

## Data Availability

The datasets used and/or analyzed during the current study are available from the corresponding author on reasonable request.

## Abbreviations

RTK: Epidermal growth factor receptor (EGFR) receptor tyrosine kinase
WT: Wild type
MD: Molecular dynamics
NSCLC: Insulin-like growth factor 1 receptor (IGF-1R) non-small cell lung carcinoma
TK: Tyrosine kinase
TKIs: Tyrosine kinase inhibitors
PDB: Protein Data Bank
CM: Comparative modeling
HRDM: High-resolution ddg_monomer
L: Leucine
R: Arginine
T: Threonine
M: Methionine
ns: Nanoseconds
CGAL: Computational Geometry Algorithm Library
RMSD: Root-mean-square-deviation
